# Simplified Surgical Conversion of Mechanical to Bioprosthetic Bentall With Leaflet Fracture Technique

**DOI:** 10.1093/icvts/ivaf277

**Published:** 2025-11-22

**Authors:** Yasuhiko Kawaguchi, Jennifer Higgins, Kassem Ashe, Gary Salasidis

**Affiliations:** Division of Cardiac Surgery, Waterloo Regional Health Network, Kitchener, Ontario, N2M 1B2, Canada; Division of Cardiac Surgery, London Health Science Centre, Western University, London, Ontario, N6A 5A5, Canada; Division of Cardiac Surgery, Waterloo Regional Health Network, Kitchener, Ontario, N2M 1B2, Canada; Division of Cardiac Surgery, Waterloo Regional Health Network, Kitchener, Ontario, N2M 1B2, Canada; Division of Cardiac Surgery, Waterloo Regional Health Network, Kitchener, Ontario, N2M 1B2, Canada

**Keywords:** aortic root replacement, redo, leaflet fracture, pannus

## Abstract

We report a case of simplified surgical conversion from a prior mechanical composite valved conduit to a bioprosthetic aortic valve using a leaflet fracture technique. A 69-year-old man presented with progressive heart failure 7 years after aortic root replacement for bicuspid aortic stenosis and root aneurysm. Imaging revealed severe prosthetic valve stenosis and suspected pannus formation. Given significant comorbidities, a simplified approach was chosen to avoid full root re-replacement. Following redo sternotomy and graft incision, the mechanical valve leaflets were fractured and removed. A Foley balloon inserted into the left ventricular outflow tract prevented leaflet embolization. Pannus excision revealed a hypertrophic subvalvular septum, prompting a septal myectomy. A 23-mm bioprosthetic valve was implanted above the retained mechanical housing using interrupted mattress sutures. The patient’s postoperative course was uneventful, and echocardiography confirmed good valve function. This case highlights the utility of leaflet fracture as a safe and efficient option in high-risk reoperative settings and underscores the added benefit of direct subvalvular visualization for detecting underlying anatomic contributors to prosthetic dysfunction not detected preoperatively.

## INTRODUCTION

Redo aortic root replacement (ARR) after a prior ARR remains technically demanding due to adhesions, coronary reimplantation, and prolonged operation times.^1^ Mechanical prosthetic dysfunction, commonly from thrombus or pannus formation, may require reintervention.^2^ Simplified alternatives, the leaflet fracture technique, have emerged to reduce procedural complexity and risk by minimizing adhesiolysis, shortening cross-clamp time. It offers a simplified surgical option, preserving the prosthetic housing, allowing for implantation of a new bioprosthesis within the existing conduit.^3^ We report a case of successful conversion from mechanical to bioprosthetic ARR using this technique, with additional pannus removal and subvalvular myectomy prompted by intraoperative findings.

## CASE REPORT

A 69-year-old man with a history of aortic root replacement with a 25-mm mechanical valved Valsalva-type conduit for bicuspid aortic valve stenosis and root aneurysm presented with progressive dyspnoea (New York Heart Association Class III). His comorbidities included prior percutaneous coronary intervention to the right coronary artery, transient ischaemic attack, atrial fibrillation, and severe chronic obstructive pulmonary obstruction with extensive smoking history. Transthoracic and transoesophageal echocardiography (TTE and TEE) revealed a significantly elevated mean pressure gradient (PG) (39 mmHg, up from 14 mmHg) across the aortic valve (AV), with an AV area of 0.9 cm^2^ without flow acceleration in the left ventricular outflow tract (LVOT), consistent with severe prosthetic valve stenosis (**Video 1**). Fluoroscopy demonstrated restricted leaflet motion (**Video 2**). Pannus-related mechanical valve dysfunction was suspected, and redo surgery was indicated. Given the patient’s comorbidities, a simplified approach—conversion to a bioprosthetic valve via leaflet fracture—was selected to reduce surgical risk.

**Figure 1.**
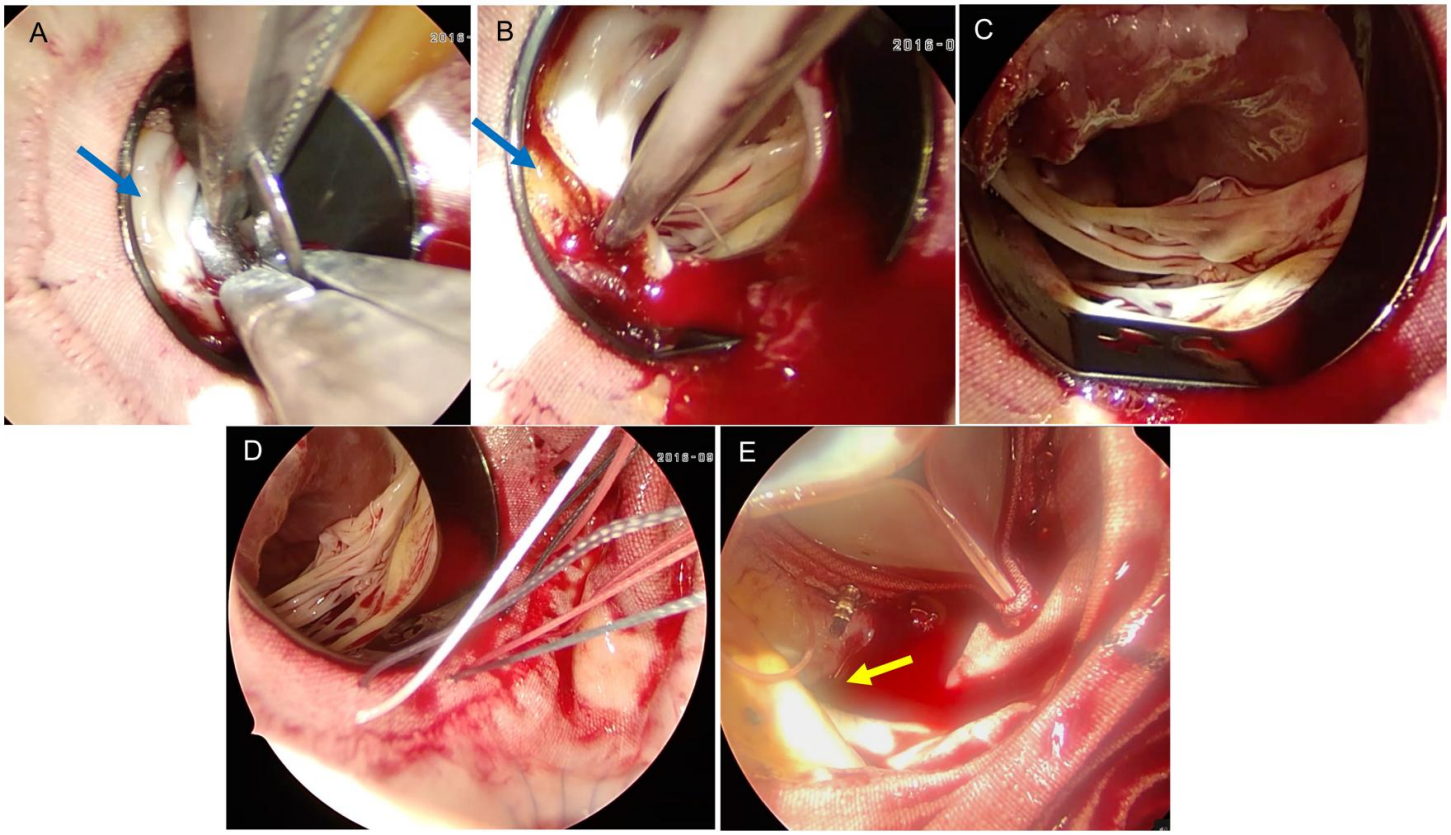
Intraoperative Findings. (A) Circumferential Pannus (arrow) Below the Mechanical Valve With an Inflated Foley catheter. (B) Hypertrophic Septum (Arrow) Below the Pannus Protruding into the Valve Housing. (C) Post-Myectomy Appearance. (D) Interrupted Horizontal Mattress Sutures Just Above the Housing. (E) Post Valve Implantation with Enough Clearance from the Left Coronary Button (Arrow)

Under the general anaesthesia, after the redo sternotomy and adhesiolysis, cardiopulmonary bypass (CPB) was established via the ascending aorta and right atrium. After aortic cross-clamping, the Dacron graft was incised, and a circumferential pannus was identified below the valve leaflets, significantly restricting their mobility (**Figure 1A**). To prevent embolism during leaflet fracture, a 14-Fr Foley catheter was inserted into the LVOT and inflated with 30 mL of saline. The mechanical leaflets were fractured and removed using 2 regular Mayo-Hegar needle drivers, and the Foley catheter was subsequently withdrawn. With the leaflets and pannus removed, a hypertrophic subvalvular septum was clearly visualized protruding into the valve housing (**Figure 1B**). Septal myectomy was performed (**Figure 1C**). Interrupted horizontal mattress 2–0 braided sutures were placed just above the mechanical valve housing. A 23-mm bovine pericardial valve was implanted in the subcoronary position and secured. Adequate coronary clearance was confirmed. CPB and cross-clamp times were 106 and 65 min, respectively (**Video 3**). Intraoperative TEE showed no paravalvular leak.

The postoperative course was uneventful. The patient was extubated on postoperative day (POD) 1 and discharged home on POD 9. Follow-up TTE showed a well-functioning bioprosthetic valve with peak and mean PG of 24.2 and 11.3 mmHg, respectively, and AV area of 1.84 cm^2^ (**Video 4**). There is no reintervention to date for 6 months.

Ethical approval was not required for a single-patient case report at our institution. Written informed consent was obtained before the publication.

## DISCUSSION

Pannus-induced prosthetic valve dysfunction is a rare but serious complication requiring surgical reintervention. The proposed mechanisms of pannus formation include non-immune inflammation to the sewing cuff, excessive tissue overgrowth due to turbulent flow, and elevated shear stress around the pivot guard. However, the precise pathophysiology remains unclear.^2^^,^^4^

In this case, we hypothesize that the patient’s hypertrophic septum and subvalvular turbulence contributed to pannus formation. Notably, this subvalvular obstruction was not appreciated preoperatively and was only identified after leaflet and pannus removal—underscoring the diagnostic advantage of this surgical approach.

Redo Bentall procedures are associated with in-hospital mortality rates ranging from 2.4% to 12%, depending on institutional experience and patient comorbidity.^1^ To reduce complexity, Vricella et al^3^ first described a simplified approach involving leaflet removal while retaining the mechanical valve housing, enabling bioprosthetic valve implantation with minimal dissection. The key techniques include using a Foley catheter to prevent embolization and placing annular sutures low and close to the valve housing to avoid coronary obstruction. Maximizing bioprosthetic valve size ensures sufficient orifice area reducing risk of flow limitation from the retained valve housing.

Our case extends this technique by incorporating septal myectomy, prompted by direct visualization through the prosthetic housing. This hybrid approach safely converted the prosthesis and addressed the underlying anatomic contributor to valve failure—findings that were not evident on preoperative imaging.

While transcatheter leaflet fracture with valve-in-valve transcatheter aortic valve replacement has been reported for inoperable patients, they carry embolic risks even with cerebral protection.^5^ This case shows the feasibility of concomitant leaflet fracture, bioprosthesis implant, and septal myectomy in a patient with the obstruction of a mechanical Bentall conduit due to pannus formation.

## Data Availability

The data underlying this article will be shared on reasonable request to the corresponding author.
